# Construction of a system for head and neck tumor traceless resection with non-inflatable transaxillary total endoscopic surgery

**DOI:** 10.1186/s12957-023-03033-6

**Published:** 2023-07-26

**Authors:** Ronghao Sun, Xu Wang, Michelle Z. Malouta, Yuqiu Zhou, Yongcong Cai, Chunyan Shui, Jian Jiang, Jianfeng Sheng, Chuanming Zheng, Wen Tian, Chao Li

**Affiliations:** 1 Department of Head and Neck Surgery, Sichuan Cancer Hospital, Sichuan Cancer Institute, Sichuan Cancer Prevention and Treatment Center, Cancer Hospital of University of Electronic Science and Technology School of Medicine, Chengdu, China; 2 Department of Psychiatry, Bloomington Meadows Hospital, 3600 N Prow Rd, Bloomington, IN 47404 USA; 3 Department of Thyroid, Head, Neck and Maxillofacial Surgery, The Third People’s Hospital of Mianyang, Sichuan Mental Health Center, Mianyang, China; 4 Department of Head and Neck Surgery, Center of Otolaryngology-Head and Neck Surgery, Zhejiang Provincial People’s Hospital, People’s Hospital of Hangzhou Medical College, Hangzhou, China; 5 Department of General Surgery, Chinese PLA General Hospital, Beijing, China

**Keywords:** Head and neck surgery, Tumor, Endoscopic surgery, Cosmetology, Transaxillary approach, Non-inflatable

## Abstract

Radical cure and functional preservation of tumors are the fundamental goals of surgical treatment of head and neck tumors, and the preservation of good aesthetics is a higher pursuit on this basis. Fully hiding the surgical incision and reducing the visibility of scars are important goals of cosmetic surgery. Using complete endoscopy for the head and neck is an effective method. CO_2_-free transaxillary total endoscopic surgery is a method with many advantages, which has been widely used in the resection of thyroid tumors, but for other parts and types of tumors in the head and neck, this surgical method is rarely used. The research team expanded its application scope and applied it to submandibular gland tumor resection and other head and neck surgeries for the first time. Through this exploration, it improved traction devices such as retractors, strictly limited the surgical indications, analyzed and summarized the key points, steps and methods of surgery, and built a treatment system for head and neck tumor surgery under complete endoscopy using the non-inflatable transaxillary approach. In this article, we introduce the system and select typical cases to share.

## Introduction

The head and neck have special complex anatomy and important physiological functions, so patients with head and neck tumors seek to cure the tumor while retaining as much of their appearance and as many important functions as possible. However, the traditional open surgery will leave obvious surgical marks and scars on the head and neck, which can seriously affect the postoperative psychology of patients through cosmetic impacts, especially among the younger population. Scar tissues can also cause some impairments to certain functions, all of which are major concerns to the patients. Endoscopy is increasingly popular among patients and surgeons because it can not only reduce the size of the incision site, but also place it in a relatively hidden location. Although endoscopic surgery technology is now relatively mature in the thoracic cavity, abdominal cavity and other parts of the body, the application of endoscopic technology in head and neck surgery lags behind due to the lack of natural lacunae and the complex anatomical relationship between the head and neck [1]. With continuing advances in science and technology, the development of improved instruments and the increasing experience level of doctors, clinicians have begun to consciously apply endoscopic surgery technology to a wider range of head and neck operations ever since Italian general surgeons first implemented thyroidectomy with endoscopic technology 25 years ago [2].

Establishing a safe and effective lumen in the distal position to reach the head and neck surgery area has always been the key to achieving head and neck endoscopic surgery. With research into basic anatomy and active exploration by surgeons in relevant specialties, a variety of approaches such as the thoraco-mammary approach, the total areola approach, the transoral approach, and the posterior auricular hairline approach have been proposed and validated [3, 4]. However, such problems as mental nerve injury and mouth angle damage after oral surgery, poor exposure of parts of the area through the posterior auricular hairline approach, and local tumor planting caused by the rupture of the sample bag of the trocar channel after thoraco-mammary endoscopic surgery make it imperfect [5]. With improvements in recent years, scholars within China have adopted the air-free suspension method through the axillary approach to achieve thyroidectomy with good surgical results. The axillary approach not only hides the incision in the axilla to avoid leaving visible scars on the neck, but also prevents some shortcomings of the approaches mentioned above [6].

Until now, however, the application of endoscopic surgery via axillary non-inflatable approach in head and neck surgery has mainly focused on thyroidectomy. In order to use this technology to achieve improved surgical treatment of head and neck tumors, our center has made continuous explorations in recent years. It has not only improved the suspension system to meet the needs of more head and neck tumor operations, but has also successfully achieved the removal of submandibular glands by the way of transaxillary non-inflatable endoscopic surgery for the first time in the world [7]. At present, we have successfully applied this method to achieve the surgical treatment of multiple types of head and neck tumors in multiple locations, including thyroid tumors, submandibular gland tumors, neck tumors, supraclavicular tumors and infraclavicular tumors. This article briefly introduces our experience and application system in the surgical treatment of head and neck tumors with transaxillary non-inflatable endoscopy.

## Surgical instruments and suspension devices

**Fig. 1:**
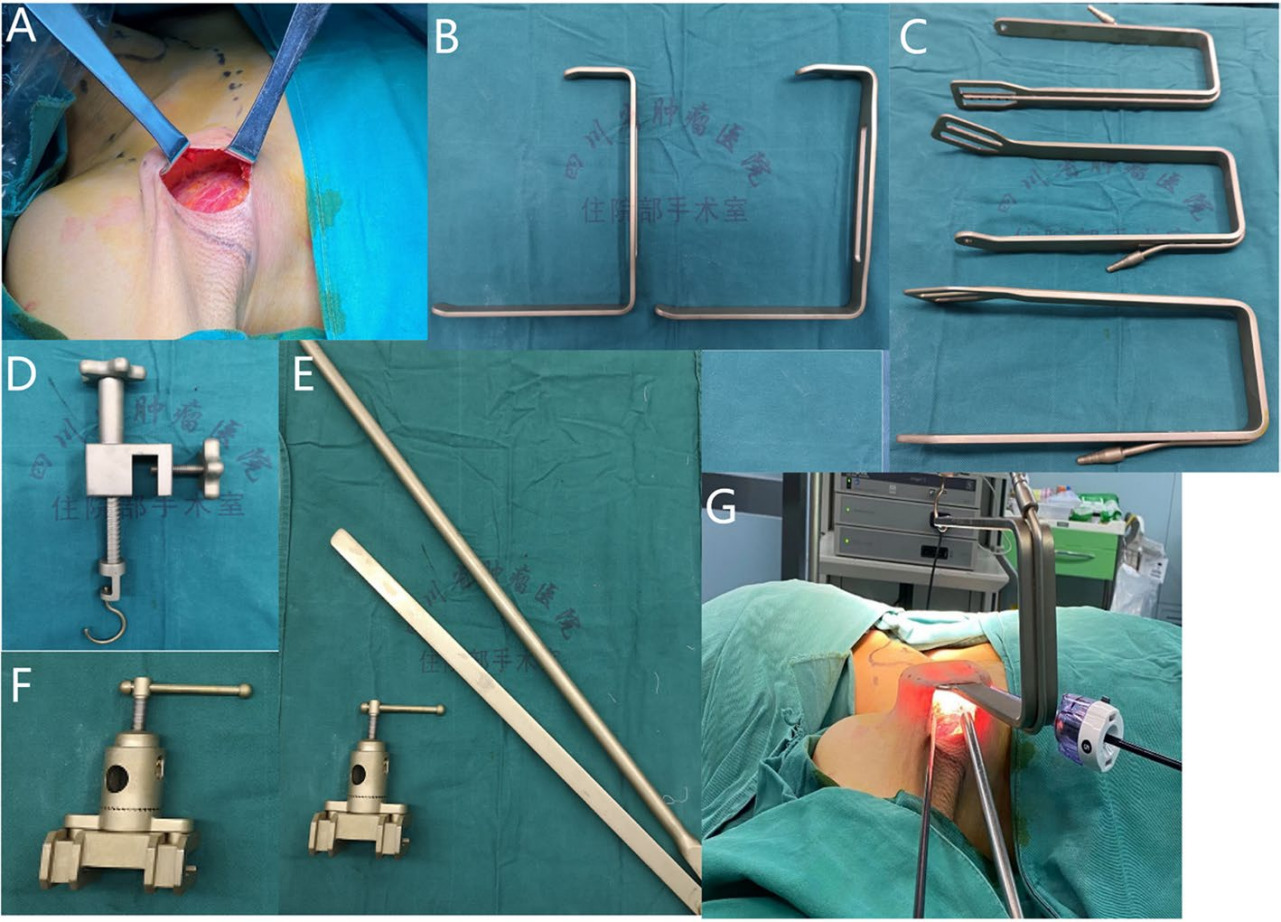
Transaxillary non-inflatable endoscopic surgical instrument and suspension device. A Pull hook for pulling skin. **B** Type II tissue hook. **C** Type I tissue hook. **D** Suspension chain winder. **E** Suspension rod. **F** Suspension fixture. **G** Intraoperative use

In order to better perform the endoscopic head and neck surgery with the transaxillary non-inflation approach, and to meet more indications for head and neck tumors, we have improved the main components such as the Chung suspension equipment and the pull hook commonly used in the past, and we have applied for the patent for this technology. To briefly introduce this set of devices:

1. The hand-held tissue puller (Fig. 1A, B) is mainly used to manually pull away the skin and subcutaneous tissue during cavity construction. Ordinary head and neck surgery pullers (such as a thyroid puller) can be selected.
2. There are three types of hanging tissue hooks (Fig. 1C), which are used in different stages of cavity building and left and right cavity building. In the initial stage of cavity building with endoscopy, we generally use straight hooks (the width of the improved front end of the hook is about 4 cm, and the length of the extended hanging wall of the hook is 20 cm). When lifting different parts of skin and muscles, we generally use left or right bending hooks. The suction device is integrated into the hooks, with smoke interference generated by energy instruments to be eliminated in time during the operation.
3. The main function of the suspension chain winder (Fig. 1D) is to fix it on the suspension rod and pull the open pull hook. During use, it should be noted that one end of the knob should face the patient’s head side, so that the patient’s foot side can have enough space to place two robot arms and reduce interference between the robot arms.
4. The suspender (Fig. 1E) is fixed beside the bed, and its function is to provide support for the suspension chain winder. When fixing the suspender, it should be placed at the level of the patient’s nose tip as much as possible, while keeping the direction of the suspender parallel to the built cavity (we have modified the suspender by reducing its height, thus improving its facilitation and increasing its stability).
5. The hanging device anchor (Fig. 1F) is used to fix the hanging rod at the bed side, adjust its height and angle to cooperate with the robot arm. The placement and use of the whole device during the operation are shown in Fig. 1G.

## Indications for head and neck surgery with non-inflatable endoscopy

**Fig. 2:**
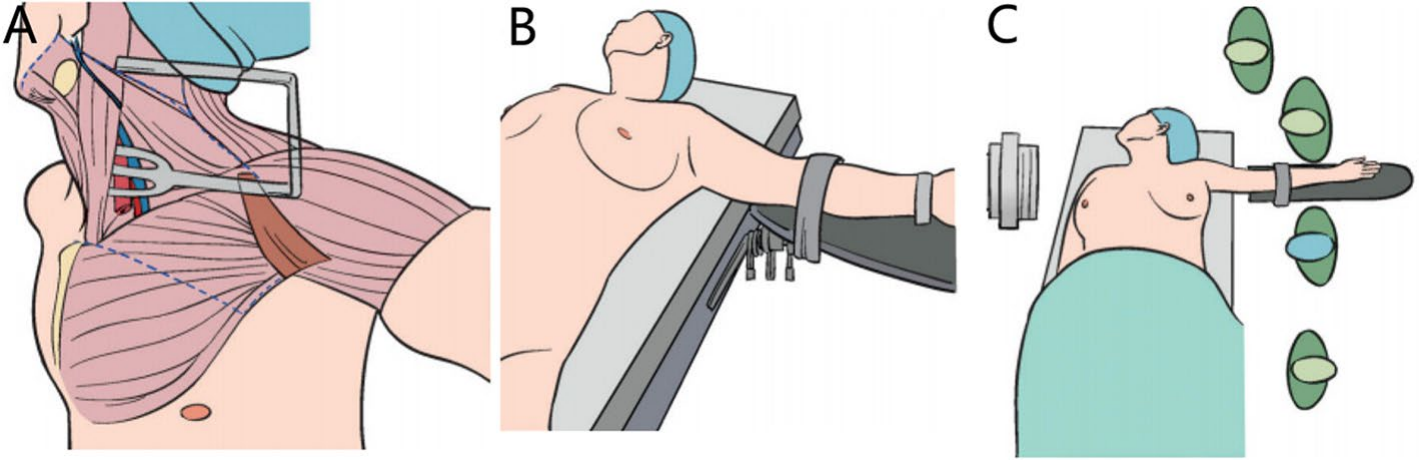
The coverage, posture and personnel standing position of the head and neck of the transaxillary non inflatable endoscopic surgery. **A** The scope that can be covered and reached in the head and neck by the axillary non-inflatable endoscopic surgery. **B** Commonly used surgical posture. **C** Schematic diagram of intraoperative operator standing positions

Through our clinical exploration and verification, the scope that can be covered by the transaxillary inflatable endoscopic surgery in the head and neck is as follows: up to the upper edge of the mandible, down to the nipple plane, inside to the medial edge of the opposite sternocleidomastoid muscle, and outside to the axillary midline (as shown in Fig. 2A). The placement of the patient's regular posture and the standing position of the operator and assistant during the operation are shown in Fig. 2B, C. If the patients have needs and meet certain surgical indications, they can use transaxillary non-inflatable endoscopic surgery to remove the tumor. However, due to the inflexibility of endoscopic surgical instruments, there are inevitable visual dead corners and tunnel restrictions in the exposure process of different parts. We believe that the application of this method should strictly adhere to the surgical indications.

Indications:

1. Benign lesions such as thyroid and submandibular gland tumors or adenomas.
2. Benign lesions such as head and neck lipomas in the upper and lower clavicle.
3. Unilateral cervical lymph node biopsy from Zone IIA to Zone IV, isolated benign tumor of the neck and neck lymph node dissection within the range.
4. Patients with hyperthyroidism requiring surgery for whom the goiter does not exceed grade II unilateral thyroid lesions.
5. The maximum diameter of benign tumor is less than 6 cm. If it is cystic, it can be relaxed to 6–8 cm.
6. Some thyroid cancers need to meet the following requirements: unilateral lesions, well differentiated pathological types with a maximum diameter of the primary focus less than 4 cm, no extraglandular invasion or only minor extraglandular invasion lesions that break through the thyroid gland anterior capsule or minor invasion of the sternal thyroid muscle, cN0 or cN1 and no mutual fusion and fixation of metastatic lymph nodes.

Relative contraindications:

1. Obesity or muscular overdevelopment;
2. Neck, chest or clavicle deformities;
3. After the tumor breaks through, the capsule or the tumor location is close to the place where the recurrent laryngeal nerve enters the larynx;
4. The metastatic lymph nodes are larger and more numerous, with envelope invasion.

Contraindications:

1. Those with serious comorbidities who cannot tolerate general anesthesia or routine surgical posture;
2. History of surgery, radiotherapy or thermal ablation on the affected side of the neck;
3. Solid benign tumor with large focus (diameter ≥ 6 cm), grade III enlarged hyperthyroidism, and retrosternal goiter;
4. Thyroid cancer with obvious extraglandular invasion, or that is accompanied by upper mediastinal lymph node metastasis or fusion and fixation of metastatic lymph nodes;
5. Thyroid cancer with poor prognosis pathological sub-type;
6. Benign tumor with self-inflammatory disease or severe surrounding inflammation.

## Selection and establishment of tunnel

Patients undergoing head and neck surgery under non-inflatable endoscopy usually adopt a supine position with their shoulders cushioned, their heads inclined to the healthy side by about 45°, and the affected side’s upper limbs naturally abducted by 90 ~ 180° (the abduction angle of the upper limbs can be adjusted appropriately according to the specific operation purpose and the patient's body shape), to expose the affected side’s armpits, to disinfect and spread towels routinely, and to make a natural fold incision along the armpits based on the preoperative design. Trocar was placed about 2 cm near the breast side of the axillary incision and slightly below the axillary front line. After the subcutaneous fat layer is cut, the long head electric knife is inserted into the gap to separate the muscle and the subcutaneous fat with the help of a hook at the insertion site. The integrity of the sarcolemma of pectoralis major should be preserved during separation. When the electrosurgical knife could not continue to separate the flap, the suspension hook was replaced, and the endoscopic separation was performed. An electrocoagulation hook or ultrasonic knife was used to separate the subcutaneous tunnel along the designated area. The pectoralis major surface flap was separated according to the surgical site to complete the establishment of the chest wall cavity.

If the tumor is in the subclavian area, the flap should be separated slightly downward without crossing the clavicle. If the tumor is located in the supraclavicular region, it needs to be separated slightly upward to build a cavity, and then separated to the vicinity of the supraclavicular fossa after crossing the clavicle to complete the establishment of the supraclavicular and inferior cavities. When separating the supraclavicular and inferior regions, pay attention to protecting the supraclavicular nerve, a branch of the cervical plexus, to reduce the numbness of the supraclavicular region after surgery. If it is a thyroid gland, submandibular gland or lateral neck tumor, it will continue to separate from the sternocleidomastoid muscle after crossing the clavicle. When it gradually separates along the surface of the sternocleidomastoid muscle, it should actively avoid or expose the external jugular vein protecting the head side.

If the tumor comes from the submandibular gland, the posterior edge of the sternocleidomastoid muscle will be exposed. Then, after separating the lateral edge of the ribbon muscle, continue to separate along the carotid triangle until the anterior and posterior abdomen of the digastric muscle is exposed, and the submandibular gland and tumor are exposed to complete the establishment of the submandibular gland cavity.

If the tumor originates from the lateral neck area, the muscle bundle of the sternocleidomastoid muscle will be separated and exposed to the deep surface across the clavicle, and the posterior edge of the sternocleidomastoid muscle will be slightly separated and exposed. The yellow and white natural muscle space or posterior edge between the sternocleidomastoid head and the clavicle is exposed naturally or after being pulled by endoscopic instruments. Separate the fascia tissue along the natural space, move the suspension hook to pull up the thoracic head or the posterior edge of the muscle, and complete the establishment of the vascular sheath. At this time, the masses (such as lymph nodes) in the lateral neck area can be completely removed, or the lymph nodes in the lateral neck area IIa–IV can be cleaned.

**Fig. 3:**
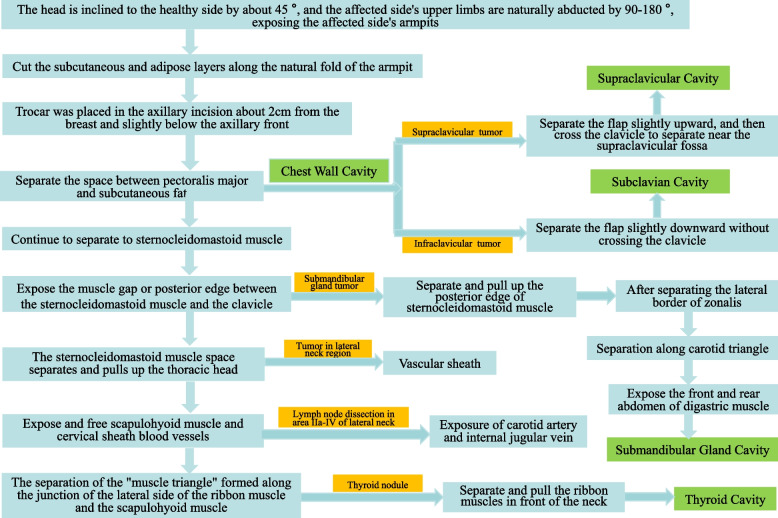
Tunnel selection and construction process for resection of head and neck tumors from different sources

If the tumor originates from the thyroid gland, the scapulohyoid muscle and the blood vessels of the cervical sheath will be further exposed and free. The scapulohyoid muscle is an important anatomical marker, and the carotid sheath and the ribbon muscle are below the muscle. The “muscle triangle” formed along the junction of the lateral side of the ribbon muscle and the scapulohyoid muscle is separated to expose the thyroid gland and complete the establishment of the thyroid cavity. See Fig. 3 for the selection and establishment of tunnels.

## Sharing typical cases

### Radical thyroidectomy

**Fig. 4:**
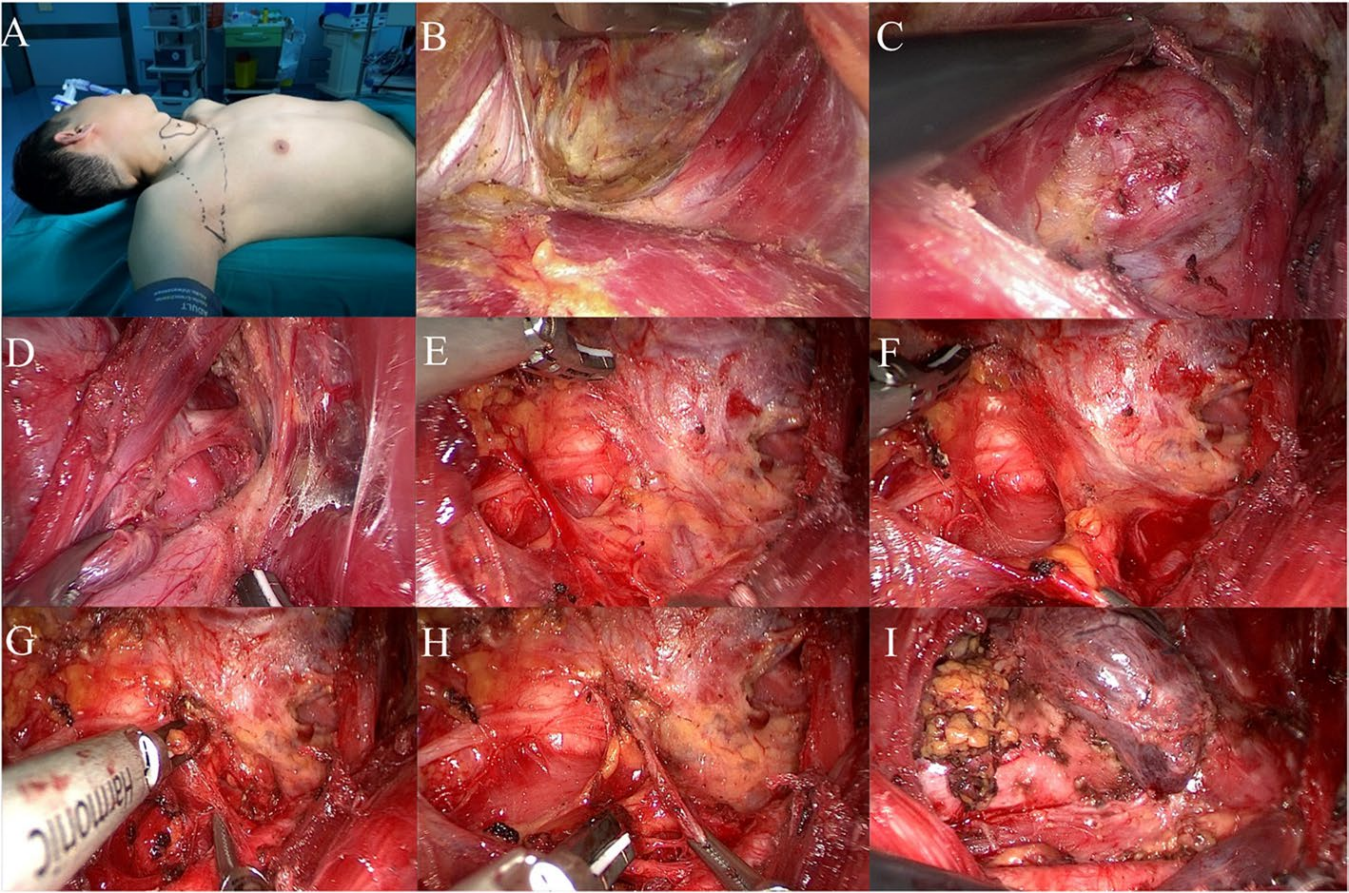
Radical thyroidectomy. **A** Surgical position and incision design. **B** Building tunnels. **C** Exposure of thyroid gland after cavity construction. **D** Bare thyroid suprapolar vessels. **E** Dissect and protect the recurrent laryngeal nerve. **F** Identify and protect superior and inferior parathyroid glands. **G** Treatment of inferior thyroid artery. **H** Lymph node dissection in the central region. **I** Complete resection of the primary focus and central lymph nodes

The patient was a young male. He was admitted to the hospital because it had been “more than 2 years since thyroid tumor was found”. Specialist physical examination: no obvious tumor was palpable in the anterior cervical region, the curve of the neck was normal, the trachea was in the middle, and no obviously enlarged lymph nodes were palpable on either side of the neck. In the auxiliary examination, the color Doppler ultrasound of the neck suggested that the middle and upper portion of the right lobe of the thyroid gland was a hypoechoic nodule with a size of about 0.5 cm × 0.6 cm × 0.5 cm, irregular shape, unclear boundary, aspect ratio greater than 1, sand-like calcification visible inside, without capsule infiltration. Cytology of the nodules showed papillary thyroid carcinoma. We improved preoperative examination and eliminated surgical contraindications. Endoscopic radical thyroidectomy plus parathyroid cell transplantation was performed under general anesthesia through the axillary approach without air inflation. The preoperative posture and identification are shown in Fig. 4A, and the tunnel is established as described above (Fig. 4B). After the construction of the thyroid cavity (Fig. 4C), the upper boundary was the upper pole of the thyroid, the lower boundary was the superior sternal fossa, the medial boundary was the junction of the opposite gland and isthmus, and the lateral boundary was the medial edge of carotid artery. As an important anatomical marker, the scapulohyoid muscle is best preserved.

The upper parathyroid gland was carefully identified in the posterior outer area of the upper pole, and the upper parathyroid gland was pulled outward and upward. The upper pole was treated by “decapitation” to expose and protect the upper parathyroid gland and its surrounding vascular network, and separated from the adjacent adipose connective tissue for protection. The position of the upper parathyroid gland was relatively fixed, with about 80–85% concentrated in a circular area with a radius of 1 cm centered on the inferior horn of the thyroid cartilage (or 1 cm above the intersection of the recurrent laryngeal nerve and the inferior thyroid artery) [8]. The surgeons separated and completely exposed the upper thyroid gland, passively separated the annular thyroid space, and exposed and bared the upper thyroid blood vessels (Fig. 4D). The ultrasonic scalpel was close to the gland to coagulate the blood vessels at multiple points. Treating the upper pole blood vessels here first can help reduce intraoperative bleeding. Meanwhile, attention had to be paid to avoid injury to the superior laryngeal nerve during operation. Intraoperative nerve monitoring equipment was used to assist in identifying and protecting the superior laryngeal nerve. The position of the inferior parathyroid gland varies greatly, and most of the time it is located in the area between the lower pole of the thyroid gland and the thymus. Therefore, its position should be judged and recognized before lymph node dissection (Fig. 4F). The adventitia of the carotid artery was dissected, and the middle thyroid vein was coagulated to expose the lymph node in the central region.

The inferior thyroid artery and the recurrent laryngeal nerve, which intersects with it vertically in the deep plane, were exposed by blunt separation in the middle and lower tracheoesophageal sulcus. Along the route of the recurrent laryngeal nerve, the surface tissue from the nerve entry point was passively separated, and the free protective nerve was dissected (Fig. 4E). The inferior thyroid artery was coagulated and cut at multiple points at the same time (Fig. 4G). After the nerve anatomy was exposed and protected, the surgeon continued to separate the lymphoconnective tissue of the lateral edge of the carotid artery from bottom to top along the esophageal muscle layer and part of the superficial surface of the prevertebral fascia (Fig. 4H). The surgeon then lifted up the lymph nodes and connective tissues in the tracheal groove as a whole and removed them along with the primary focus and the ipsilateral glands (Fig. 4I). The tumor was removed from the specimen bag and frozen during the operation: papillary thyroid cancer. The surgical team then washed and stopped bleeding, placed the plasma drainage tube, and sutured the axillary incision. The patient’s intraoperative bleeding was 5 ml, and the operation time was about 62 min. The postoperative pathology was papillary thyroid carcinoma. The patient was discharged 2 days after operation with a total drainage volume of 30 ml. After follow-up for more than 5 years, the incision was grade A healed, and no hoarseness, numbness of hands and feet, numbness in the anterior cervical region (Fig. 10A), swallowing disorder or other symptoms were found. There was no residual tumor or recurrence sign by color Doppler ultrasound.

### Submandibular gland resection

**Fig. 5:**
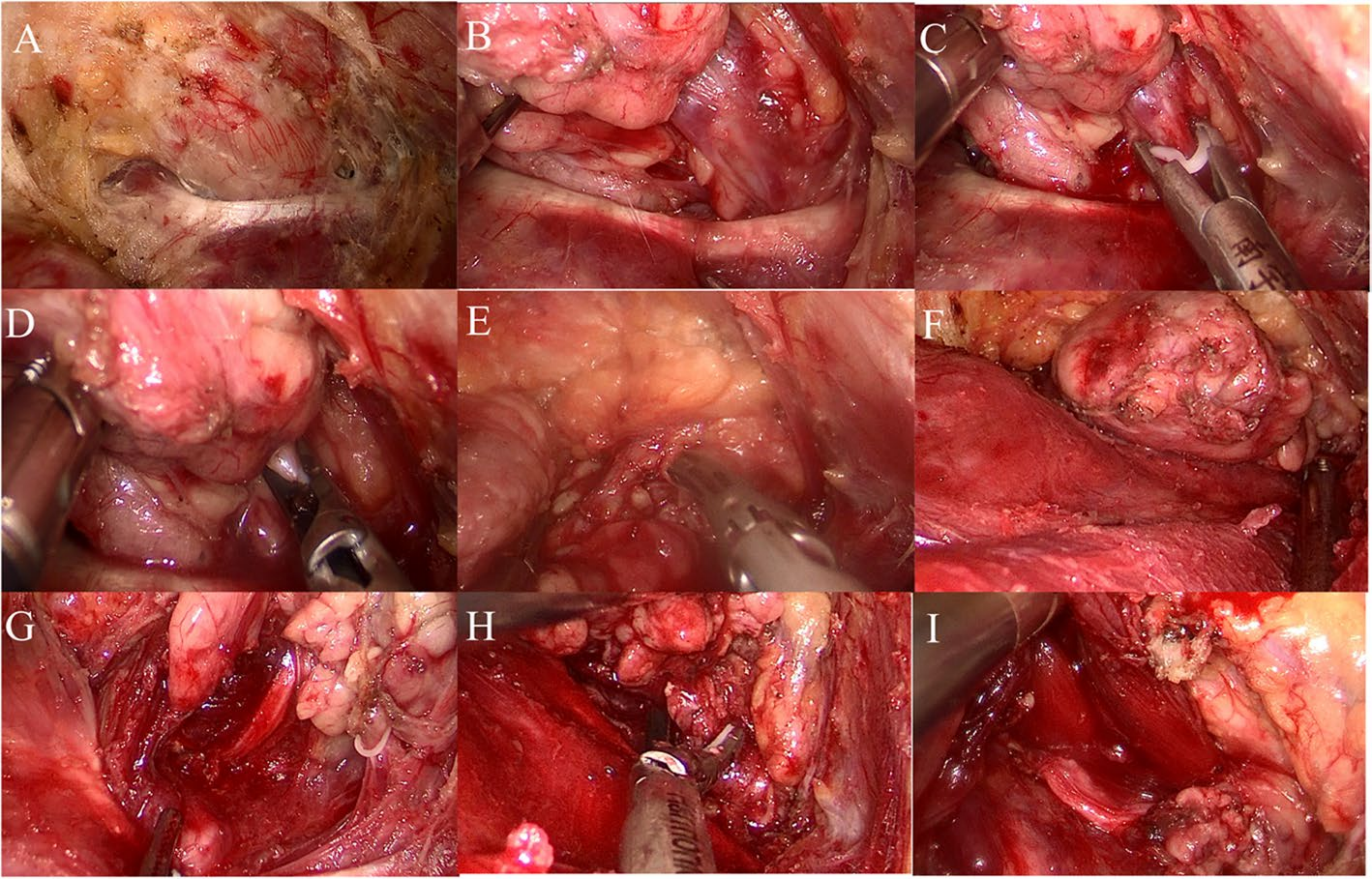
Submandibular gland resection. **A** Free digastric muscle, exposed submandibular gland. **B** The posterior edge of the free submandibular gland showed the proximal end of the artery. **C** Proximal superior artery clamp of completely free facial artery. **D** The proximal posterior segment of the facial artery was severed by multi-point coagulation. **E** The distal end of the artery appeared on the front edge of the free submandibular gland. **F** Fully free the lateral margin of submandibular gland and tumor. **G** The lingual nerve was exposed from the medial margin of the free submandibular gland. **H** The submandibular gland duct was isolated and disconnected after multi-point coagulation. **I** Hypoglossal nerve can be seen in the operation area after tumor resection

The patient, a middle-aged female, was admitted to the hospital because she had “found a mass in the right submandibular area for more than 39 years”. Specialist physical examination: a round mass with a size of about 3 cm can be touched in the right submaxillary area, soft in nature, with moderate mobility, without redness, swelling and tenderness. Color ultrasound showed a low echo mass of the right submandibular gland, about 2.1 cm × 1.6 cm × 2.1 cm in size, regular shape and clear boundary. CT showed that the right submandibular gland occupied a space, about 1.9 cm × 2.0 cm in size, tending towards benign lesions. Under general anesthesia, the submandibular gland tumor was resected by non-aerated transaxillary approach. If the transaxillary tunnel is established before, the area from the neck to the submandibular gland is separated obliquely upward after passing through the clavicle. During the operation, the submandibular gland is exposed by the free digastric muscle (Fig. 5A). It can be seen that the tumor is located behind the gland without invasion outside the gland. The proximal end of the facial artery was exposed at the posterior edge of the free submandibular gland (Fig. 5B), the proximal end of the facial artery was clamped by the deep abdominal vessel clamp behind the digastric muscle (Fig. 5C), and the proximal end of the facial artery was severed after multi-point coagulation (Fig. 5D). The whole deep surface of submandibular gland can be dissociated.

The submandibular gland was pulled downward to passively separate between the upper edge of the submandibular gland and the lower edge of the mandible, and the mandibular marginal branch of the facial nerve was anatomically protected.

Free the front edge of the submandibular gland, find and show the distal end of the artery, and coagulate with an ultrasonic knife (Fig. 5E). Continue to free the upper part of the submandibular gland, fully free the lateral edge of the submandibular gland and the tumor (Fig. 5F), expose the lingual nerve at the medial edge of the free submandibular gland, anatomize and protect the lingual nerve (Fig. 5G), free the submandibular gland duct and disconnect it after multi-point coagulation (Fig. 5H), and finally completely remove the submandibular gland and the tumor (Fig. 5I). Take out the tumor from the specimen bag, wash and stop bleeding, place the plasma drainage tube, and suture the axillary incision. The patient’s intraoperative bleeding was less than 10 ml, and the operation time was about 90 min. The postoperative pathology was pleomorphic adenoma. The patient was discharged on the 3rd day after operation with a total drainage volume of 110 ml. After a follow-up of 1 year, the incision of the patient showed grade A healing, without facial paralysis, neck and upper limb pain or movement disorder, neck skin sensation reduction or numbness, and oral saliva secretion reduction (Fig. 10B).

### Supraclavicular tumor resection

The patient, a middle-aged female, was admitted to the hospital for “finding the right supraclavicular mass for 3 years”. Specialist physical examination: about 3 cm mass was palpable in the right supraclavicular area, with medium texture, clear boundary and moderate mobility. No pain, redness, ulcer, etc. Lipoma was considered after conducting the routine preoperative examination. Under general anesthesia, the supraclavicular tumor was resected by transaxillary gas-free endoscopy.

**Fig. 6:**
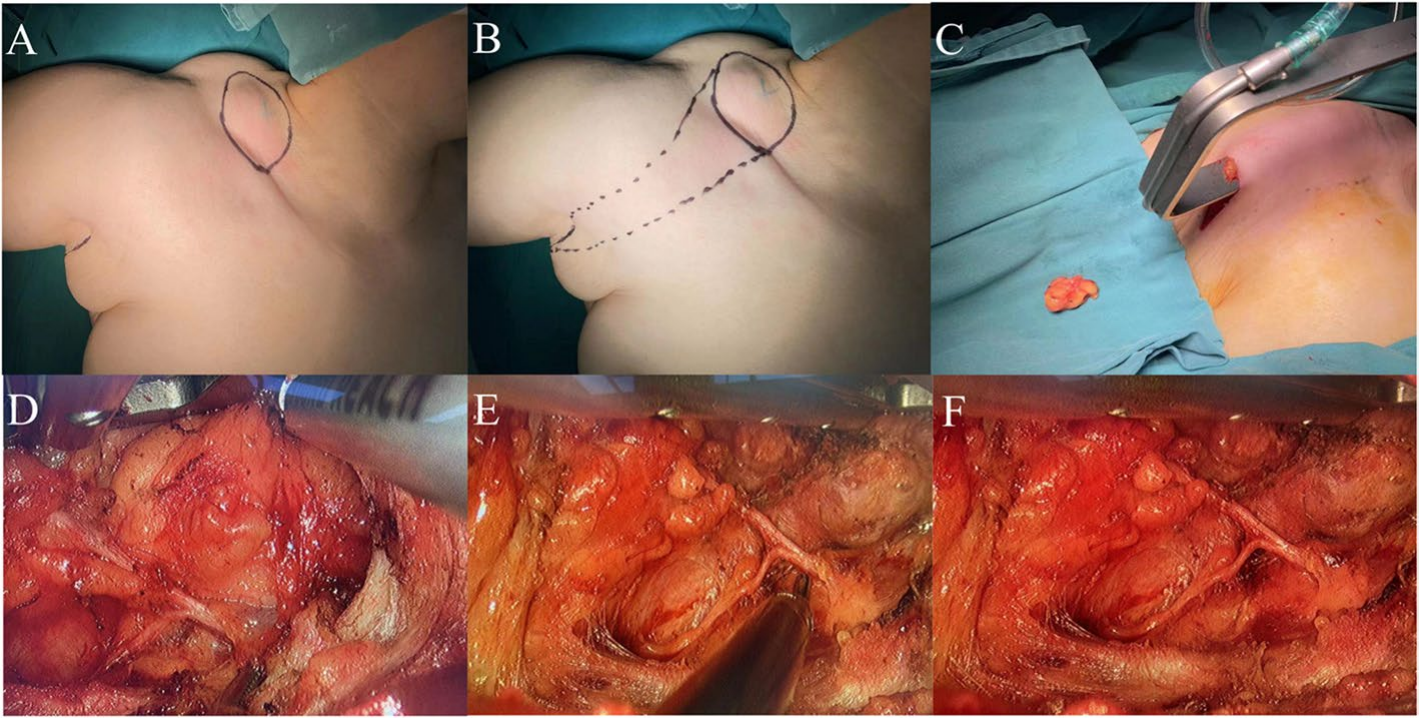
Supraclavicular tumor resection. **A** Location of supraclavicular mass. **B** Preoperative identification. **C** Building tunnels. **D** Completely free supraclavicular mass. **E** Protect the supraclavicular epithelial nerve. **F** Surgical area after tumor resection

The procedure was as follows: design the direction, width and length of the tunnel before operation (Fig. 6A, B). For example, make an axillary wrinkle incision in the front, separate it along the subcutaneous tissues, and then use a special hook to establish a tunnel and a surgical cavity (Fig. 6C). When it is separated to the supraclavicular area, it can see a fat mass with a clear boundary. Separate it around the tumor with an ultrasonic knife (Fig. 6D), pay attention to protecting the supraclavicular epithelial nerve (Fig. 6E), and remove it after completely peeling off the mass (Fig. 6F). Follow-up for 1 year showed no special findings (Fig. 10C).

### Excision of tumor in subclavian region

The patient, a young female, was admitted to the hospital for “10 years since the tumor under the right clavicle was found”. Physical examination: the neck is soft, the trachea is in the middle, and a tumor of about 5 cm is palpable in the area below the middle of the right clavicle. The texture is soft, the boundary is clear, and the mobility is fine. No pain, redness, ulcer, etc. After improving the routine preoperative examination, lipoma was still considered in the last case before operation. Similarly, under general anesthesia, the tumor in the subclavian area was resected under the axillary air free endoscope.

**Fig. 7:**
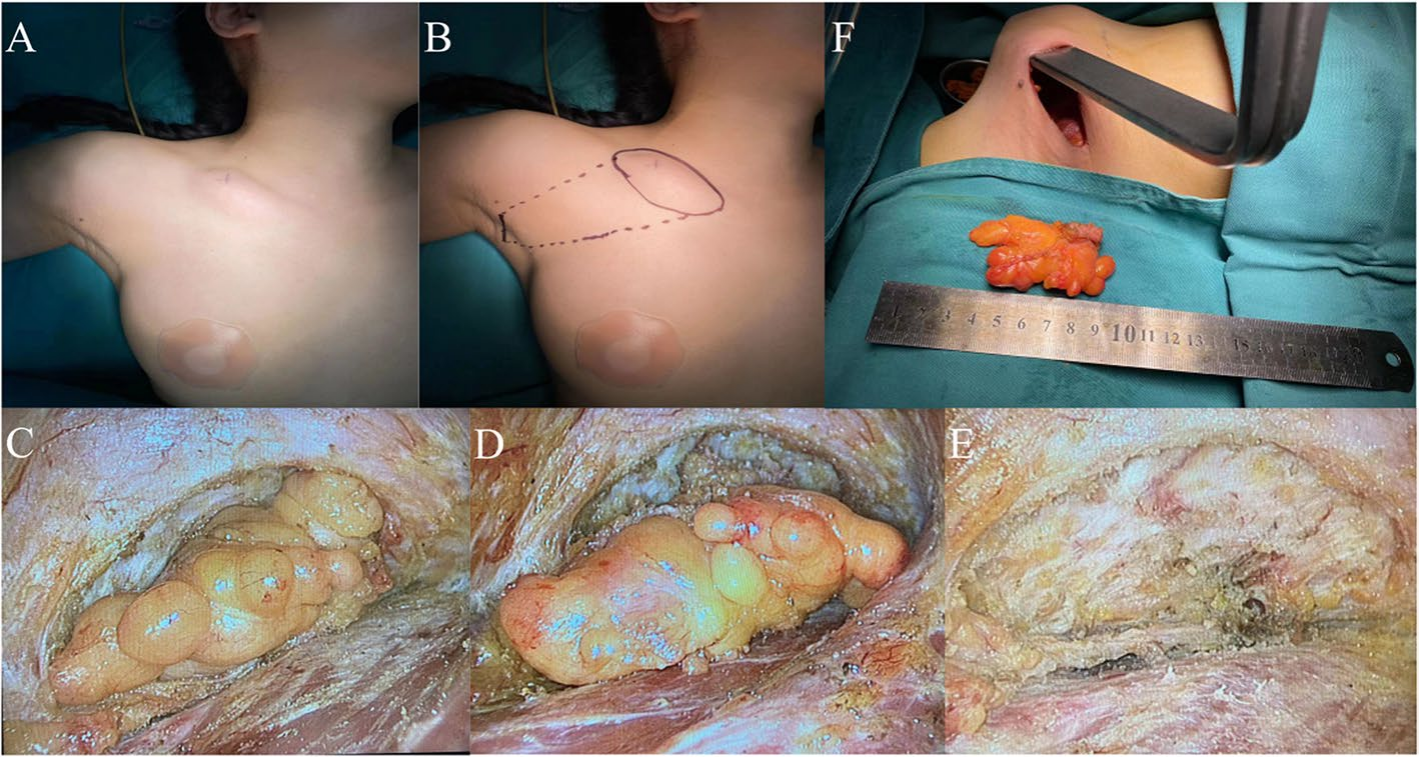
Excision of tumor in subclavian region. **A** Location of tumor in subclavian area. **B** Preoperative identification. **C** Separate the tumor in subclavian area. **D** Completely free the tumor in the subclavian area. **E** Surgical area after tumor resection. **F** Special drawhook for cavity construction and tumor removal

The procedure was as follows: Design the direction, width and length of the tunnel before operation (Fig. 7A, B). As mentioned above, the skin flap will not be separated upward until it is separated to the subclavian area through the axilla. Then, a tunnel and a surgical cavity will be established with a special hook (Fig. 7F). After fully freeing the surrounding tissue of the chest wall, the tumor can be seen as shown in Fig. 7C, with clear boundary, lobulated shape and light yellow color. It will be separated by an ultrasonic knife along the tumor. After completely freeing the tumor (Fig. 7D), it will be completely removed (Fig. 7E). After resection, the operation area is shown in the figure (Fig. 7E), and the tumor is taken out through the axillary tunnel with a specimen bag (Fig. 7F). Status of the patient's surgical area after 1 year of follow-up was shown in Fig. 10D.

### Neck lymph node biopsy

The patient, a young female, was admitted to the hospital because “1 year after thyroid cancer surgery, a tumor in the right neck area was found 3 months ago”.

**Fig. 8:**
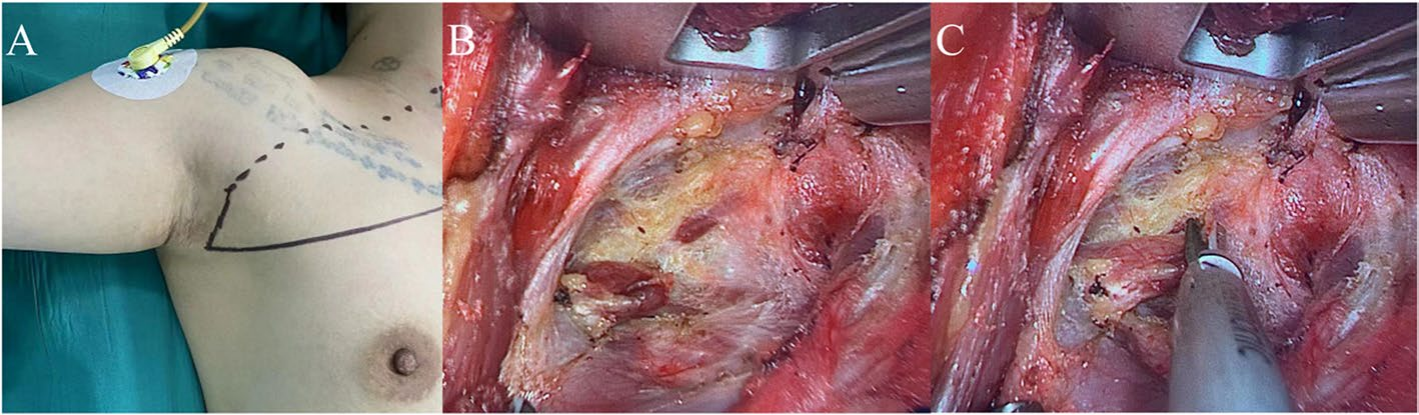
Neck lymph node biopsy. **A** Cervical lymph node location and preoperative identification. **B** The lymph nodes in Area III of the lateral neck were separated. **C** Completely dissociate and resect the lateral cervical region III lymph nodes

Physical examination: a mass with a size of about 2.5 cm was palpable in Area III of the right neck, with hard texture, unclear boundary and fair mobility. Color Doppler ultrasound showed enlarged lymph nodes in Area III of the right neck, unclear boundary between skin and medulla, and abnormal aspect ratio. Considering the possibility of lymphohematopoietic system diseases, biopsy is recommended. Then, under general anesthesia, cervical lymph node biopsy was performed under endoscope through the axillary approach without air inflation. The preoperative posture, neck lymph node location and preoperative identification are shown in Fig. 8A. The surgeon cut the subcutaneous and adipose layers along the natural fold of the armpit, separated the space between the pectoralis major muscle and the subcutaneous fat, and then continued to separate to the sternocleidomastoid muscle, exposing the muscle space between the sternocleidomastoid muscle’s sternal head and the clavicle bone. The surgeons then separated and pulled up the sternocleidomastoid muscle’s space along the sternocleidomastoid muscle, exposing the enlarged lymph nodes in Area III of the right neck (Fig. 8B), protect the neck sheath, free and completely remove the lymph nodes in Zone III of the lateral neck with an ultrasonic knife (Fig. 8C). The wound cavity was rinsed, a plasma drainage tube was placed, and the axillary incision was sutured with absorbable thread. The patient was discharged one day after operation with a total drainage volume of 10 ml. Postoperative pathology: diffuse large B cell lymphoma. There were no special complications or discomfort in the operation area. After a 3-year follow-up, the patient’s surgical incision and area recovered well (Fig. 10E).

### Lateral neck selective (level IIa–IV) lymph node dissection

**Fig. 9:**
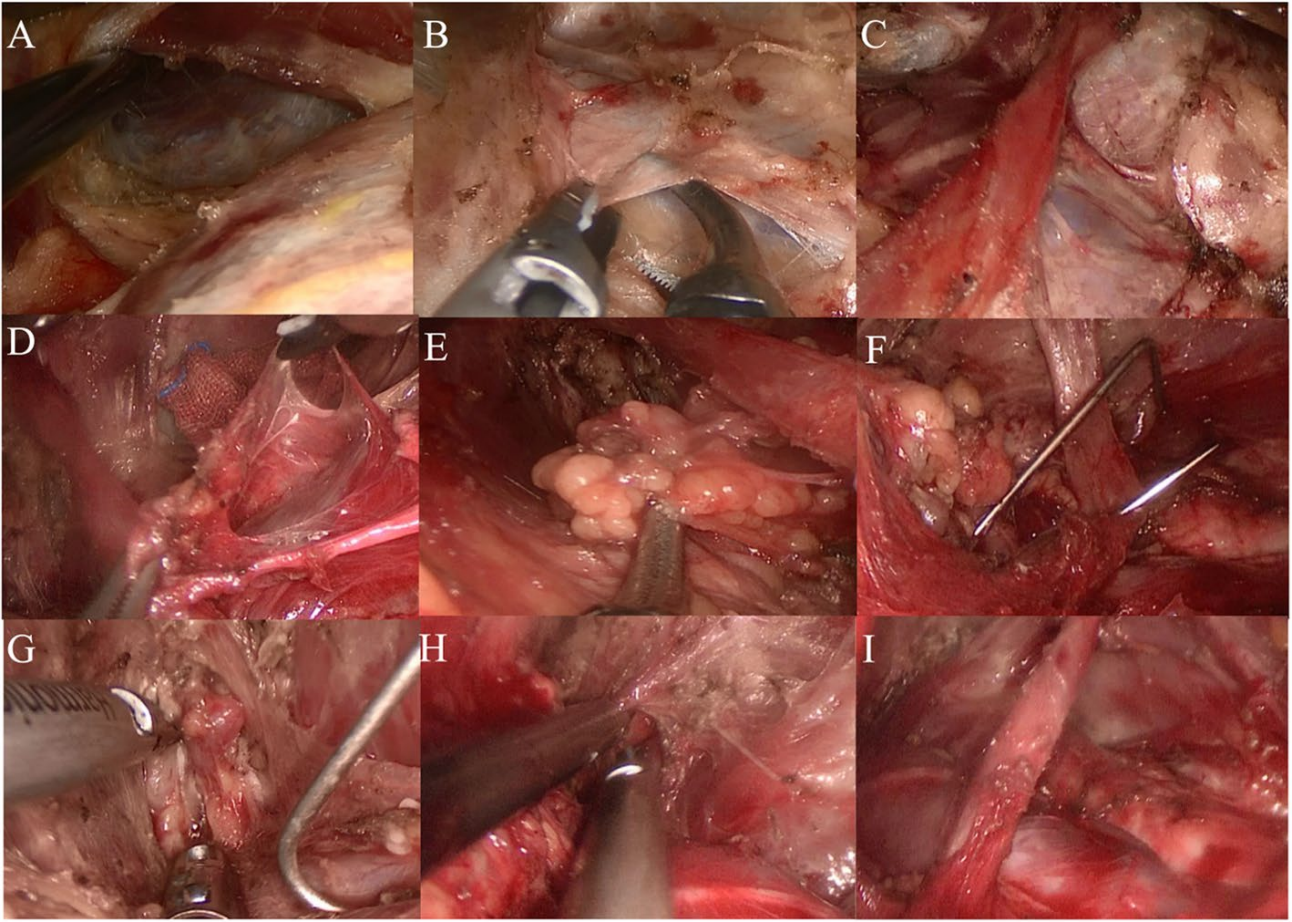
Lateral neck selective (level IIa–IV) lymph node dissection. **A** Free posterior edge of sternocleidomastoid muscle. **B** The internal jugular vein was exposed by free cervical sheath. **C** Free and retain the scapulohyoid muscle. **D** Dissection of lymph nodes in Area IV. **E** Dissection of lymph nodes in Zone III. **F** Implant a pull hook to pull the internal jugular vein. **G** Dissection of lymph nodes in Area IIa. **H** Expose the digastric muscle. **I** Operation area after lymph node dissection of right cervical region IIa–IV

**Fig. 10:**
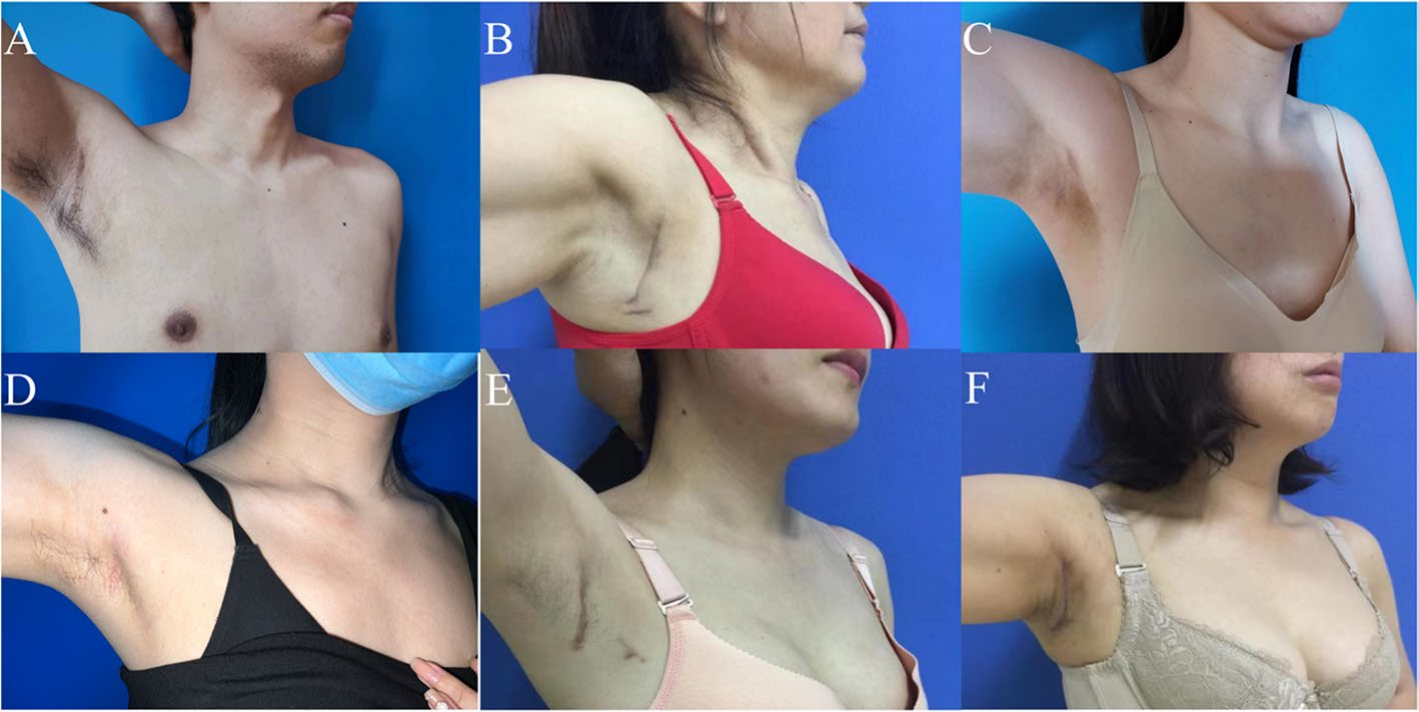
Postoperative photos of the last follow-up of the 6 cases who undergoing CO2-free transaxillary total endoscopic surgery that mentioned above. **A** 5 years after thyroid cancer surgery. **B** 1 year after resection of submandibular gland tumor. **C** 1 year after resection of supraclavicular tumor. **D** 1 year after resection of tumor in subclavian region. **E** 3 years after lymph node biopsy. **F** 2 years after lymph node dissection

The patient, a middle-aged female, was admitted to the hospital for “had been found the right neck tumor for more than 3 months”. Three years ago, due to thyroid cancer (BRAF gene mutation), the patient underwent total thyroidectomy + double central area lymph node dissection via the thoraco-mammary approach under complete endoscopy in the external hospital. She was followed up regularly after surgery. Three months ago, color Doppler ultrasound found that the lymph nodes in Areas III and IV of the right neck were enlarged, with a size of about 1.5–2.0 cm, unclear boundary, and unclear boundary between skin and medulla. Metastasis is considered. Lymph node cytology showed malignant tumor. Specialist physical examination: the neck is soft, the trachea is in the middle, the thyroid gland on both sides is untouched, the swollen lymph nodes can be reached in Areas III and IV of the right neck, the texture is hard, the mobility is fine, and there is no redness, swelling or tenderness. The preoperative examination was improved, and after the exclusion of surgical contraindications, right cervical selective (Area IIa–IV) dissection was performed under general anesthesia through the non-inflatable transaxillary approach. The transaxillary tunnel was established as shown in the previous Fig. 3. After confirming and protecting the supraclavicular epithelial nerve, it was separated to the sternocleidomastoid muscle with an electric hook or an ultrasonic knife. The posterior edge of the sternocleidomastoid muscle was freed, with attention paid to protecting the external jugular vein (Fig. 9A). After the hook entered the lower space, it was pulled up to expose the cervical sheath. The free cervical sheath exposed the internal jugular vein (Fig. 9B). The surgical team separated and retained the omohyoid muscle (Fig. 9C). First, the surgeons cleaned the lymph nodes in Zone IV from the lower part of the hyoid scapula muscle to the trunk of the head and arm (Fig. 9D), then gradually separated and dissected upwards. During the dissection, attention must be paid to identifying the descending branch of the hypoglossal nerve, completely cleaning the lymph nodes in Zone III from the upper part of the hyoid scapula muscle to the plane of the cricoid cartilage (Fig. 9E), and fully exposing Zone IIA after implanting a needle-like draw hook to pull and compress the internal jugular vein. The accessory nerve was separated and dissected to protect it, the lymph nodes in Area IIA in front of the accessory nerve were completely cleaned until the digastric muscle at the upper boundary of area II was exposed (Fig. 9F–H). The deep branches of the cervical plexus were dissected and protected, and the lymph nodes and adipose tissue in Zone IIA–IV were completely removed along the surface of the anterior fascia of the vertebra. The specimen bag was taken out and sent for postoperative medical examination. The surgical area was rinsed, and hemostasis was achieved. After cleaning, the operation area (Fig. 9I) showed a skeletal state, indicating that all the important anatomical structures are completely preserved. The plasma drainage tube was placed, and the axillary incision was sutured. The patient’s intraoperative bleeding was about 45 ml, and the operation time was about 96 min. The postoperative pathology was metastasis of thyroid papillary carcinoma. The patient was discharged 3 days after operation with a total drainage volume of 90 ml. There were no short-term complications such as bleeding. After 2 years of follow-up, the incision of the patient healed grade A, without long-term complications, neck and upper limb pain or movement disorder, and no symptoms such as decreased skin sensation or numbness in the neck (Fig. 10F).

## Discussion

Because of its special position, head and neck surgery not only affects the function of patients, but also seriously affects their appearance. Doctors in head and neck surgery have been seeking to cure diseases while retaining the appearance in order to improve patient satisfaction. The results of many studies in which patients report outcomes consistently show that with comparable treatment effects, patient satisfaction should be an important indicator for clinical treatment decision-making and measurement of treatment effects [9, 10]. The exposure of the surgical incision, scar formation and the accuracy of repair and reconstruction will all affect the satisfaction of patients after head and neck surgery. In recent years, transferring the head and neck incision to a hidden area away from the neck, which effectively avoids the impact of surgical incision and postoperative scar on the appearance and achieves an ideal aesthetic effect, is an important breakthrough in head and neck surgery technology and concept.

The way to achieve this change is to perform surgery in a complete endoscopic manner. In the past, due to the particularity and complexity of head and neck anatomy, most clinicians thought that it was not appropriate to perform complete endoscopic surgery in the head and neck where there was no natural anatomical cavity or important anatomical structures gathered. For tumor patients, especially patients with malignant tumors, complete endoscopic surgery cannot achieve the same radical effect as open surgery [11–13]. These factors once hindered the application of total endoscopic surgery to the head and neck. However, with the progress of technology, some head and neck doctors have begun to try to operate with complete endoscopy, and the most common procedure is thyroidectomy under complete endoscopy. Studies have confirmed that for some patients who meet the criteria for surgery, thyroidectomy under complete endoscopy is safe and feasible compared with open surgery [14]. In addition, the endoscope has the advantages of enlarging the surgical field of view to improve the surgical accuracy and the recognition of important micro-anatomical structures [15]. As technology advances, many older approaches are gradually eliminated, and new approaches are constantly developed and tested. Finally, the three most commonly-used surgical approaches under complete endoscopy in head and neck surgery are mainly transaxillary, transoral, and transthoracic. The cosmetic effect of the transoral approach is good, but the operation is the most complex and difficult among the three approaches [16]. The operation of the transthoracic mammary approach is relatively simple, but scars can still be formed around the chest wall and areola, and the aesthetic effect is noticeable [17]. However, the transaxillary approach combines the advantages of the above two approaches and avoids their disadvantages. In particular, the non-aerated transaxillary approach avoids the risk of CO_2_ entering the blood and causing gas embolism. Thus, it has gradually gained the attention of head and neck surgeons and become more popular [18].

Some scholars used the non-aerated transaxillary approach to perform radical surgery for thyroid cancer under endoscopy. Compared with open surgery, there was no significant difference in the scope of central lymph node dissection and the thoroughness of surgery [19]. Similarly, after comparison, scholars found that this method can effectively reduce the possibility of accidental injury to blood vessels and significantly reduce the amount of bleeding. Moreover, the total complication rate is no different from that of open surgery, but the types of complications are obviously different. The complications of endoscopic surgery through axillary approach are mainly manifested as skin flap damage and postoperative skin congestion, which may be related to the damage of blood supply of the flap caused by an excessively thin free skin flap or subcutaneous congestion during the operation. However, through immediate measures to improve circulation and reduce local activities, they gradually improve after a few days and basically disappear at discharge. The main complications of open surgery were recurrent laryngeal nerve and parathyroid gland injury. However, the degree of neck and chest pain and the overall satisfaction with aesthetic appearance after radical thyroidectomy through the axillary approach under the endoscope without inflation are significantly better than that of open radical thyroidectomy [20]. Therefore, based on the review of a large number of current studies, the results all show that for some patients who meet the criteria after strict screening (such as unilateral thyroid microcarcinoma at cN0 stage), the choice of complete endoscopic surgery, especially the transaxillary approach, has become a safe and effective choice, in addition to being the best aesthetic one.

Since the non-inflatable transaxillary approach can be effectively applied to thyroid surgery, can it be applied to more head and neck surgery? In clinical practice, our team found that using this surgical method can achieve the resection of a wide range of head and neck tumors from the lower edge of the mandible to the nipple plane, from the lateral side to the mid axillary line, and from the medial side to the medial edge of the opposite sternocleidomastoid muscle (see Fig. 2A). However, if we want to achieve the above range of tumor resection and establish a complete treatment system by using the non-inflatable transaxillary endoscopic surgery, we first need to improve the existing traction device. Therefore, we have improved the type I tissue retractor (Fig. 1C), first narrowing the width of the pulling surface at the front end, which is more conducive to putting into the tissue gap of the head and neck. Then, we processed the edge of the U-shaped hook handle in an arc shape, which can effectively avoid the damage caused by the relatively thin skin of the head and neck when pulled by the hook. In addition, in order to reach further anatomical parts (such as submaxillary area), we also lengthened the type I tissue retractor. At the same time, a negative pressure suction device is integrated into the pull hook to reduce the interference of intraoperative smoke to the surgical process, and it can also be used as a water source interface for irrigation of the surgical area. Finally, in terms of materials, a more solid nickel chromium alloy is selected. The length and material of the same suspension rod have also been similarly improved (1-E). Other devices such as a type II tissue puller and suspension chain winder retain the current design of thyroid surgery and only optimize the materials to strengthen their flexibility.

Secondly, it is more important to strictly grasp the surgical indications in the non-aerated transaxillary approach cavity lens neck tumor surgery system. The existing research, compared with open surgery, is based on strict patient screening, which can be carried out only under the condition of meeting local requirements and is applicable to most head and neck tumor operations. In clinical application, we must be careful in expanding the indications. In the system we have built, the above indications and contraindications are mainly based on existing research results, evidence-based medical evidence and our experience. With the accumulation of more evidence and experience, it is believed that the surgical indications of the non-inflatable transaxillary lens neck tumor surgery will improve over time.

Thirdly, the reason why this technique can form a head and neck tumor treatment system is that it has some of the same surgical steps and methods, and has similarities in some surgical techniques and precautions, such as the methods and steps of cavity construction. Once these common parts are mastered, it will be conducive to the development of various operations. However, there are similarities as well as differences, and different surgical paths need to be taken according to the type and location of specific tumors. Among them, the selection and construction of tunnels and surgical cavities are particularly important. We summarize them in Fig. 3 for your learning and reference, hoping to help doctors in relevant departments make correct decisions. We successfully carried out the world’s first non-inflatable endoscopic submandibular gland resection via axillary approach [7] by using this system, and verified its effectiveness and safety through follow-up with a number of patients. Of course, for the removal of tumors in the upper and lower clavicles, selective neck lymph node dissection and other operations, we have also used the non-inflatable transaxillary approach under endoscopy many times, achieving satisfactory clinical results. We have also shared the surgical data of typical cases in the relevant section of this article. The patients’ satisfaction and quality of life were followed up with the Chinese version of the European Organization for Research and Treatment of Cancer Quality of Life Questionnaire (EORTC QLQ-C30) [21].

Based on this, we believe that the surgical indications, special instruments, operating methods and surgical steps described in the non-inflatable transaxillary approach cavity lens neck tumor surgery system can achieve cosmetic resection of head and neck tumors. Compared with conventional open surgery and other approaches of endoscopic surgery, it has the following advantages:

1. It can meet the requirements of radical tumor surgery, the incision is hidden, and there is no scar on the neck after surgery. It achieves a good cosmetic effect by making an incision at the natural fold of the armpit skin. There is no scar on the neck or chest.
2. Good postoperative comfort: the operation is entirely carried out within the potential space of the neck, so the skin and flesh of the patient are not separated during the operation, the muscles are not broken, and there is no neck discomfort or chest tightness after the operation [19].
3. No need to fill CO_2_ gas: most conventional endoscopic operations need to fill CO_2_ gas to assist in cavity construction and maintain the surgical space. There are still related complications such as subcutaneous emphysema, mediastinal emphysema, CO_2_ accumulation in blood and tissues, and even rare gas embolism that leads to patient death. However, endoscopic thyroid surgery via axillary approach does not need to be filled with CO_2_ gas, and special hooks are used to assist in cavity construction and maintain the surgical space. There is no worry of carbonation during the operation, no risk of gas embolism, and simple anesthesia management [22].
4. Endoscopy has a magnification effect of 5–10 times, which can more carefully and thoroughly clean the central and lateral lymph nodes. The important tissue structure was clearly revealed during the operation to avoid surgical injury.
5. For patients who have undergone plastic surgery in the maxillofacial region or breast region, or those who have high aesthetic requirements for the above-mentioned region, this surgical method can still be used for neck traceless endoscopic surgery.
6. Small trauma, fast postoperative recovery, no numbness, foreign body feeling and swallowing linkage [23].
7. The operation space is large, the main knife and the assistant are convenient to cooperate, and the posture of the operator is relatively comfortable during the operation.
8. The resected specimen is easy to take out, and it is still applicable to head and neck tumors with relatively large volume.

Nevertheless, according to our experience, we still need to pay attention to some details in the operation to improve the success rate of the operation and reduce postoperative complications. For example, when performing radical thyroidectomy with transaxillary non-inflatable endoscopy, pay attention to coagulating the inferior thyroid artery and its surrounding veins as far as possible away from the trachea to ensure paraglandular blood supply. It is difficult to expose the lymph nodes at the thoracic entrance, and the “chopstick effect” is serious, which requires continuous adjustment and change of the endoscopic instruments and lens positions. Clean the anterior tracheal lymph nodes, protect the upper end of the thymus or the lingual lobe of the thymus, and prevent the incorrect cutting of ectopic paraglands in the thymus or blood supply damage to the inferior paraglands [24, 25]. At the same time, we should also pay attention to the hidden lymph nodes below the thymus. Generally, the anatomical variation of the inferior paraglandular area is large and it is not easy to preserve. For parathyroids that cannot be preserved in situ, cannot be guaranteed good blood supply or are accidentally removed, the strategy of parathyroid auto-transplantation should be decisively adopted. Free transplantation should be carried out in the pectoralis major muscle near the incision and marked with non-absorbable suture [26]. In addition, after the ipsilateral glands and lymph nodes in the central region are completely separated and lifted, the principle of bipolar electrocoagulation and re-cutting can be considered for treatment of the thyroid suspensory ligament area at the entry point, so as to minimize thermal injury, bleeding and complete removal of glands. If electrical instruments are used for operation, the suspensory ligament should be disconnected once as much as possible, and multiple disconnections may cause residual glands. Saline gauze can block the entry point of the recurrent laryngeal nerve and reduce thermal injury. If the tissue at the suspensory ligament is relatively dense, it can also be separated layer by layer with an electrocoagulation hook. Finally, it is also necessary to use the specimen bag to take out the tumor from the axillary incision in whole, and take diligent care so as to avoid planting and metastasis of the tumor.

It should be pointed out that although this method has been widely evaluated in the surgery of thyroid tumors, it is still in our trial and exploration stage for other head and neck tumors, including submandibular gland tumors. At present, the number of cases is limited, and it is impossible to carry out a large sample of case–control or randomized controlled trials to verify its safety and effectiveness in other head and neck tumor surgery. The current assessment only depends on our previous experience. At present, we have initially established this system. In the future, we will further collect cases for control trials to comprehensively evaluate its safety, effectiveness, and cosmetic.

In summary, by improving the surgical hook device, specifying the surgical indications and contraindications, reasonably planning the surgical steps, and practical clinical application and verification, we have built a set of transaxillary non-inflatable cavity lens neck tumor surgery system suitable for head and neck tumor resection. This system completely summarizes the possibility and method of non-inflatable endoscopic surgery via axillary approach for head and neck tumors, and provides a system of traceless surgical treatment for head and neck tumors. After strict case screening, the full and reasonable use of this method can meet the aesthetic needs of patients on the premise of achieving radical cure of tumors and preservation of functions. We have verified that it has clinical feasibility and effectiveness and significantly improves patient satisfaction. Of course, this system needs to be further improved, which requires the research and application of more scholars and doctors to provide more abundant and powerful evidence. It is hoped that this system will be recognized by more head and neck surgeons and colleagues in the clinic, which will help the technical development and improvement of head and neck surgery, and also benefit more head and neck tumor patients with aesthetic needs.

## Data Availability

The datasets used or analyzed during the current study are available from the corresponding author on reasonable request.
